# Factors Associated with Patient's Delay in Tuberculosis Treatment in Bahir Dar City Administration, Northwest Ethiopia

**DOI:** 10.1155/2014/701429

**Published:** 2014-05-20

**Authors:** Endalew Gebeyehu, Muluken Azage, Gedefaw Abeje

**Affiliations:** ^1^Department of Pharmacology, College of Medicine and Health Sciences, Bahir Dar University, P.O. Box 79, Bahir Dar, Ethiopia; ^2^Department of Public Health, College of Medicine and Health Sciences, Bahir Dar University, P.O. Box 79, Bahir Dar, Ethiopia

## Abstract

*Background*. Unknown proportions of tuberculosis cases remain undiagnosed and untreated as result of several factors which further increases the number of tuberculosis cases per index case. *Objective*. To identify factors associated with patient's delay in initiating treatment of tuberculosis. *Methods*. Cross-sectional study was employed from January to April, 2013, in Bahir Dar Ethiopia. A total of 360 patients were included. Data were collected from tuberculosis patients using a semistructured questionnaire. Data were entered and analyzed using SPSS version 16 windows. Multivariate logistic regression analysis was used to identify factors associated with patient delay. *Results*. Of all patients, 211 (62%) sought medical care after the WHO recommended period (21 days). The median patient delays of smear positive, smear negative, and extrapulmonary patients were 27 (IQR: 10–59), 30 (IQR: 9–65), and 31 (IQR: 10–150) days, respectively, with statistically significant variations among them (ANOVA: *F* = 5.96; *P* < 0.003). Place of residence and educational status were the predictors of patient delay. *Conclusion*. Around two-thirds of all patients and more than half of smear positive tuberculosis patients were delayed in seeking medical care within the recommended period. Provision of DOTS service in the vicinity and health education on TB may reduce patient delay and its consequences.

## 1. Introduction

Tuberculosis (TB) is a major public health burden throughout the world. Almost one-third of the world population (about 2 billion) is infected with* Mycobacterium tuberculosis* and during the past decade even industrialized countries have faced a resurgence of tuberculosis. Currently, TB is the leading cause of mortality among infectious diseases worldwide. About 95% of TB cases and 98% of deaths due to TB occur in developing countries [1].

Ethiopia is one of the 22 countries with high TB burden [1]. Literatures showed that many TB cases remain undiagnosed and untreated in many countries with high incidence of TB that increases the risk of TB transmission in the community [1–8]. Studies on different parts of the world showed that different factors are associated with patient delay. Studies done in African countries mentioned that residence was an important predictor of patient delay in initiating treatment [9–13]. Similarly in Asia and African countries being female was mentioned as a factor for predicting patient delay [9, 10, 13–15].

According to the WHO Global TB report, 2012, Ethiopia ranks 13th in the list of 22 high burden countries (HBCs) and 4th in Africa, with an estimated prevalence of all forms of TB in 200 per 100,000 population [16]. Although treatment is available freely, patients are not able to initiate treatment as early as possible. In 2011, of all causes of death in the Amhara region TB was ranked 7th for adult death. Health facility reports showed that patients failed to seek medical care within the recommended period [17].

There is paucity of credible evidence on the extent of delay in treatment seeking behavior of patients in Amhara region. Credible evidence on predictors of patient delay could be important information to look for new strategy to curb the death and spread of TB. Therefore this study aims to investigate the extent of patient delay and its influencing factors among tuberculosis patients in Bahir Dar town. Moreover, the data from this study will identify barriers for delay in care seeking to strengthen the strategies for prevention and treatment of TB patients.

## 2. Methods and Materials

### 2.1. Study Design and Period

Institution based cross-sectional study was conducted on tuberculosis patients at DOTS in public health facilities from January to April, 2013.

### 2.2. Study Setting

The study was conducted in Bahir Dar town which is the capital city of Amhara National Regional State. It is located at 565 km northwest of Addis Ababa, Ethiopia. The town has a population of 221,991 of whom 49% were male and the rest were female [18]. There were nine public healthcare facilities (one hospital and eight health centers) in Bahir Dar city administration at the time of the study. All public health institutions were providing smear microscopy and DOTS service to the community [19]. Bahir Dar town was selected because of its more numbers of clinics that conduct TB diagnosis through smear microscopic examination and DOTS service in the region to get the required sample size.

### 2.3. Sample Size and Sampling Procedures

The sample size was determined using the formula for estimation of population proportion (*n* = [(*Z*_*α*/2_)^2^∗*p*∗(1 − *p*)] /*d* ^2^) where: *Z*_*α*/2_ at 95% CI (1.96), *d* marginal error 0.05, and *p* proportion obtained from previous study in East Wollega, Ethiopia, in 2006 (0.63)[20]. With 5% none response rate, the total sample size of the study was 376. All adult tuberculosis patients registering for DOTS service in the clinics were the study population. Adults who were unable to respond and children were excluded from the study because they may not give appropriate responses to the questionnaire. All patients registering for DOTS were interviewed. To avoid double counting of patients when they change their DOTS clinic, reminder note was attached with the referral sheet to the receiving health facility.

### 2.4. Data Collection Tools and Quality Assurance

Data were collected using a semistructured questionnaire which has a question of sociodemographic and economic variables, accessibility, and availability of TB service and delay question. The questionnaire was prepared in English first and translated to the local language, Amharic and again back translation to English was made to ensure the consistency of the questions. In addition, pre test was done in an area different from the study area to validity the questionnaire. In this study, patient delay was measured by considering the time from initial onset of sign and symptoms of the disease to first consultation of healthcare facility by the patient. According to WHO recommendation the study subjects were grouped into those who delayed ≤21 days and those delayed >21 days and this binary classification was used to identify factors related to patient delay during logistic regression analysis [21]. The completeness of responses to the questionnaires was checked routinely during data collection. Questionnaires with incomplete response were excluded during data entry.

### 2.5. Data Analysis

Questions were coded and then data were entered and analyzed using SPSS version 16. Frequency runs, cross-tabulations, and sort were checked for data quality management. Descriptive statistics were used to summarize data and tables and figures were used to display results. Adjusted odds ratio with the combination of confidence interval and *P* value was calculated to identify factors associated with delay in seeking treatment using bivariate and multivariate logistic regression analysis.

### 2.6. Ethical Clearance

Ethical approval was obtained from Bahir Dar University Ethical Clearance Committee. Permission was taken from Bahir Dar City Health Office and the respective health facilities. Informed verbal consent was obtained from study participants. The study participants were assured that the data were used only for the study purpose and could not be passed to a third party. Moreover, privacy and confidentiality were maintained by giving codes for the questionnaire.

## 3. Results

Of the total 376 study participants, 360 (96%) were included in the study, whereas the remaining were excluded due to data incompleteness during data analysis. Of the study participants, 59.4% were males, 33.9% lived in rural areas, and 34.2% were illiterate. Fifty-eight percent of the study participants had extrapulmonary TB. Two-thirds of the study participants lived within 10 Km radius to public health facility (Table 1).

### 3.1. Patient Delay

The median patient's delay of smear positive, smear negative, and extrapulmonary patients was 27 (IQR: 10–59), 30 (IQR: 9–65), and 31 (IQR: 10–150), respectively. The overall median patient delay was 30 days (IQR: 10–96, and the 10th and 90th percentiles were 2 and 385 days, resp.). The maximum patient delays in smear positive, smear negative, and extrapulmonary TB patients were 365, 380, and 3650 days, respectively. Ninety-one patients (25.3%) and 24 (6.7%) came after 90 days and one year, respectively. The mean (±SD) patient delay in smear positive, smear negative, and extrapulmonary TB patients was 56 (102), 64 (98), and 192 (467) days, respectively. In ANOVA analysis patient delay had shown a statistically significant variation by categories of TB (*F* = 5.96; *P* < 0.003). A total of 211 (61.7%) patients sought medical service after 21 days which is out of the range recommended by WHO. Of all delays, 52% of smear positive and 57.7% of smear negative patients and 61.4% of extrapulmonary TB patients sought medical advice after 21 days (Table 2).

**Figure 1:**
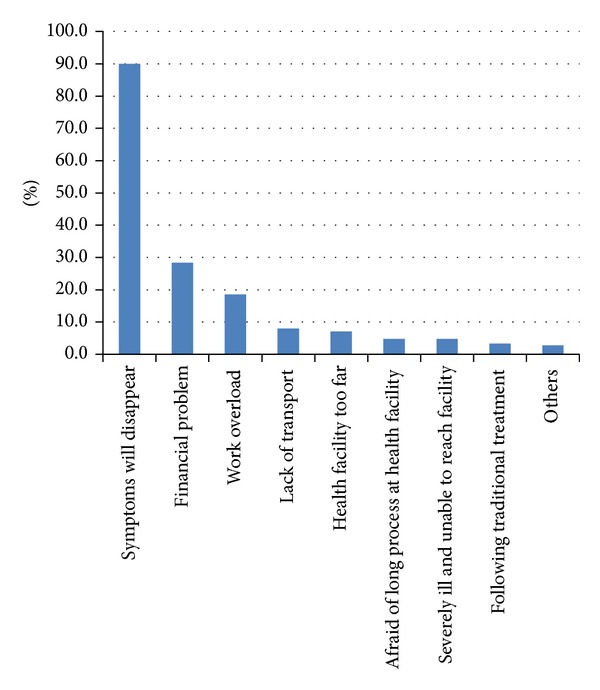
Reasons for patient delay among TB patients in public health facilities of Bahir Dar city administration, Northwest Ethiopia (there were multiple responses).

The most frequent reasons mentioned by those who sought treatment after 21 days were thinking that symptoms will disappear (90%), financial problem (28.4%), work overload (18.5%), transport problem (8.0%), health facility being too far (7.1%), being afraid of a long process at health facility (4.7%), being severely ill and unable to reach (4.7%), on traditional treatment (3.3%), and others (2.8%) (Figure 1).

### 3.2. Factors Associated with Patient Delay

Analysis result of bivariate regression indicated that place of residence, education status, and occupation (farmer and student) were statistically significant association with patient delay (Table 3). But only education status remained statistically significant (*P* < 0.05) after controlling confounding effect with multivariate regression by entering those variables that have a *P* value of ≤0.2. Illiterate patients were 3.73 times {AOR: 3.73, 95% CI (1.87, 7.44)} more delayed when compared to patients with college and above educational status. Similarly, patients with eight to twelve grade educational status were 2.74 times {AOR: 2.74, 95% CI (1.36, 5.49)} more delayed when compared to patients with college and above educational status (Table 3).

## 4. Discussion

Early detection of cases and treating TB patients are one of the strategies of WHO to reduce the diseases morbidity and mortality throughout the world. The overall median patient delay in this study was 30 days. This finding is lower than studies done in Ethiopia (60–63 days) [4, 22, 23] and other countries, Nepal (50 days) [24] and Tanzania (120 days) [12]. However, it is almost consistent with another study done in Ethiopia (28 days) [20]. The median patient's delays of smear positive, smear negative, and extrapulmonary patients were 27, 30, and 31 days, respectively, which is lower than the median patient delays for smear positive (90 days), smear negative (60 days), and extrapulmonary tuberculosis patients (90 days) done in Tigray region, Ethiopia, in 2001 [23]. The discrepancies may be due to accessibility of healthcare facilities since more than two-thirds of participants in this study are living in urban setting. Moreover, it may be the contribution of implementation of health extension programme which promotes healthcare activities in Ethiopia.

In this study, 58% of the study participants were extrapulmonary TB which is higher than the study done in Ethiopia, Tigray (39%) [23] and WHO report 2012 (32%) [16]. This difference may be due to the flow of high proportion of referral and nonreferral patients for availability of extrapulmonary TB diagnosis facility at Bahir Dar where the referral hospital is located.

In this study, educational status of below college level was an independent predictor for patient delay. Illiterate patients and patients with eight to twelve educational status are 3.73 and 2.74 times more likely delay when compared to patients with college and above educational status, respectively. This could be due to the fact that those patients who had education status of college and above level have better information access about TB and more likely to seek medical care from healthcare facilities at early stage of the diseases. Similar findings were reported in previous studies [25, 26]

This study has a limitation of recall bias since patient delay was measured by asking about initiation of sign and symptoms retrospectively. Due to the nature of the cross-sectional study design underreporting or overreporting of patient delay is unavoidable.

## 5. Conclusions

In this study, more than sixty percent of TB patients sought treatment after WHO recommended periods (21 days) and educational status was the predictor of patient delay. Therefore, provision of health education on TB should be strengthened to reduce patient delay.

## Figures and Tables

**Table 1:** Characteristics of tuberculosis patients in DOTS clinic of public healthcare facilities in Bahir Dar city administration, Northwest Ethiopia.

Variables	Frequency (*n* = 360)	Percent
Sex		
Male	214	59.4
Female	146	40.6
Age		
<20	39	10.8
20–39	243	67.5
≥40	78	21.7
Place of residence		
Urban	238	66.1
Rural	122	33.9
Marital status		
Single	152	42.2
Married	168	46.7
Widowed	14	3.9
Divorced	26	7.2
Education status		
Illiterate	124	34.2
Read and write	38	10.6
Elementary	56	15.6
Junior/secondary	80	22.2
College and above	62	17.5
Occupation		
Employee	42	11.7
Merchant	24	6.7
Farmer	73	20.3
Daily laborer	57	15.8
Student	65	18.1
Housewife	99	27.5
Smoking habit		
Yes	17	4.7
No	343	95.3
Type of tuberculosis		
Pulmonary	153	42.5
Extrapulmonary	207	57.5
Distance to the nearest healthcare facility		
<10 km	270	75
≥10 km	90	25

**Table 2:** Patient delay by TB categories in Bahir Dar city administration, Northwest Ethiopia, 2013.

	Smear positive *N* (%)	Smear negative *N* (%)	Extrapulmonary TB *N* (%)	Total *N* (%)
Patient delay in days (≤21 days)				
1–7 days	16 (21.3)	19 (24.4)	46 (22.2)	81 (22.5)
8–14 days	10 (13.3)	11 (4.1)	18 (8.7)	39 (10.5)
15–21 days	10 (13.3)	3 (3.8)	16 (7.7)	29 (8.1)
Total	**36 (48.0)**	**33 (42.3)**	**80 (38.6)**	**149 (38.3)**
Patient delay in days (>21 days)				
22–30 days	8 (10.7)	9 (11.5)	23 (11.1)	40 (11.3)
31–60 days	14 (18.7)	15 (19.2)	20 (9.7)	49 (13.6)
61–90	5 (6.7)	7 (9.0)	19 (9.2)	31 (8.6)
>90 days	12 (16.0)	14 (17.9)	65 (31.4)	91 (25.3)
Total	**39 (52.0)**	**45 (57.7)**	**127 (61.4)**	**211 (61.7)**

**Table 3:** Logistic regression analysis of factors associated with patient delay in public health care facilities of Bahir Dar town, Northwest Ethiopia.

Variables	Patient delay		COR (95% CI)	AOR (95% CI)
	≤21 days	>21 days		
Sex				
Male	87	127	1.08 (0.70, 1.65)	
Female	62	84	1.00	
Age				
<20	19	20	0.56 (0.26, 1.22)	
20–39	103	140	0.72 (0.42, 1.22)	
≥40	27	51	1.00	
Place of residence				
Urban	114	124	1.00	1.00
Rural	35	87	2.29 (1.43,3.65)**	1.74 (1.04, 2.90)
Marital status				
Single	65	87	1.00	
Married	65	103	1.18 (0.76, 1.85)	
Widowed	6	8	1.00 (0.33, 3.01)	
Divorced	13	13	0.75 (0.33, 3.79)	
Education level				
Illiterate	38	86	4.75 (2.47,9.15)***	3.73 (1.87,7.44)***
Read and write	15	23	3.22 (1.39,7.46)**	2.58 (1.08,6.15)*
Elementary	20	36	3.78 (1.76,8.11)**	3.39 (1.57,7.33)**
Junior/secondary	34	46	2.84 (1.42,5.68)**	2.74 (1.36,5.49)**
College and above	42	20	**1.00**	**1.00**
Occupation				
Employee	26	16	1.00	
Merchant	12	13	1.92 (0.70, 5.31)	
Farmer	20	53	4.31 (1.92,9.66)	
Daily laborer	25	32	2.08 (0.92, 4.69)	
Student	23	42	2.97 (1.33,6.63)	
Housewife	44	55	2.03 (0.97, 4.25)	
Smoking habit				
Yes	8	9	0.79 (0.85, 1.99)	
No	141	202	1.00	
Distance to the nearest health institutions				
<10 km	114	156	1.00	
≥10 km	35	55	1.15 (0.71, 1.87)	

*P-value < 0.05, **P-value < 0.01, ***P-value < 0.001.

## Abbreviations

AIDS: Acquired immunodeficiency syndrome
AOR: Adjusted odds ratio
CI: Confidence interval
COR: Crude odds ratio
DOTS: Direct observed treatment short course
HIV: Human immunodeficiency virus
MDG: Millennium development goal
MTB: Mycobacterium tuberculosis
SPSS: Statistical packages for social science
TB: Tuberculosis.
